# Contemporary antiretroviral pharmacology in the male genital tract: implications for HIV treatment and prevention

**DOI:** 10.1128/aac.01401-25

**Published:** 2026-03-23

**Authors:** Noah C. Neverette, Jordan Winfield, Nathalia Chaparro-Cáceres, Ken S. Ho, Julie B. Dumond, Aaron S. Devanathan

**Affiliations:** 1 Center for Clinical Pharmaceutical Sciences, Department of Pharmacy & Therapeutics, School of Pharmacy, University of Pittsburgh 6614, Pittsburgh, Pennsylvania, USA; 2 Barry and Judy Silverman College of Pharmacy, Nova Southeastern University 44255, San Juan, Puerto Rico; 3 Division of Infectious Diseases, School of Medicine, University of Pittsburgh 6614, Pittsburgh, Pennsylvania, USA; 4 Division of Pharmacotherapy and Experimental Therapeutics, UNC Eshelman School of Pharmacy, University of North Carolina 2331, Chapel Hill, North Carolina, USA; Samsung Medical Center Department of Infectious Diseases, Seoul, Republic of Korea

**Keywords:** antiretrovirals, HIV, pharmacokinetics, semen, seminal plasma

## Abstract

The male genital tract (MGT) plays a critical role in HIV transmission and persistence, serving as a viral reservoir and potential site of HIV compartmentalization. While antiretroviral therapy effectively suppresses systemic viral replication, drug penetration into the MGT tissues and semen varies considerably between and within drug classes, potentially leading to subtherapeutic concentrations in the MGT. The organs comprising the MGT have anatomical barriers and express several drug-metabolizing enzymes, resulting in immune privilege, active drug efflux, and site-specific metabolism. These factors may collectively limit antiretroviral efficacy in this compartment. This review discusses the evidence of HIV persistence in MGT organs and provides insight into the anatomical, physiological, and pharmacological considerations to target this viral reservoir. Additionally, we synthesize current knowledge on contemporary antiretroviral pharmacokinetics within the MGT, highlighting differences in drug penetration between and within classes. We identify associations between drug properties (e.g., lipophilicity and protein binding) and distribution into the MGT. Finally, we forecast emerging therapeutic approaches that introduce new pharmacological opportunities and challenges, as well as advanced computational techniques to help us better understand the downstream effects of antiretroviral pharmacology at the site of action. Understanding the interplay between antiretroviral penetration, local inflammation, and viral dynamics is essential for optimizing HIV treatment and prevention strategies. Together, these insights set the stage for targeted studies to guide precision dosing based on local therapeutic exposure thresholds. Addressing remaining knowledge gaps in MGT pharmacology will be essential to overcome anatomical and physiological barriers and achieve sustained viral suppression in the MGT.

## INTRODUCTION

Antiretroviral therapy (ART) effectively suppresses HIV in the blood plasma (BP) (1). However, cessation of ART results in viral rebound in the BP (1). Viral rebound occurs because HIV persists in body tissues—known as viral reservoirs—where HIV-infected CD4^+^ T cells, macrophages, and dendritic cells can survive and produce replication-competent virus (2, 3). HIV tissue reservoirs include the central nervous system, lymphoid tissues, gastrointestinal-associated lymphoid tissues, and the genitourinary tract; these represent the main barriers to an HIV cure (2). HIV persistence promotes chronic inflammation, leading to HIV-associated comorbidities and an increased risk of developing drug-resistant viral strains (1, 2).

Importantly, the male genital tract (MGT) and semen are viral reservoirs (3). Semen is the main vector of HIV transmission (4). Over 90% of new HIV cases reported in the United States were acquired during male-to-male or heterosexual sexual contact (5). HIV RNA has been detected in the semen of people with HIV (PWH) with BP viral loads <200 copies/mL (6). The HIV RNA viral dynamics differ between seminal plasma (secretions from male reproductive glands; SP) and BP; HIV RNA in SP can fluctuate independently of that in BP (6). Additionally, the virus in the MGT and semen may become genomically dissimilar to the virus in the BP, termed compartmentalization (7). Together, independent viral dynamics and compartmentalization within the MGT and semen provide strong evidence of local viral replication in this compartment (6, 8). Furthermore, compartmentalization may result in the development of drug-resistant HIV strains, promoting viral egress from the semen into the blood following ART cessation (9, 10). Following egress, proliferation, and replication of drug-resistant strains occur, leading to widespread, permanent drug resistance (11). Therefore, consistent adherence to ART is vital for preventing the development of multidrug-resistant HIV (12).

Viral persistence in the MGT also leads to localized HIV-associated inflammation, the degree to which is directly associated with viral load in the semen (13). This represents a feedback loop: Localized inflammation leads to the recruitment of immune cells susceptible to infection, driving enhanced viral replication and resulting in further localized inflammation (13–15). This feedback loop may increase the risk of HIV transmission (16). Therefore, understanding the mechanisms underlying HIV-associated inflammation in the MGT allows for more accurate prediction and prevention of HIV transmission for PWH on ART.

Inadequate distribution of antiretrovirals (ARVs) into the MGT and SP may facilitate the development of the viral reservoir (2, 17). Penetration of ARVs into the semen varies between drug classes and between ARVs in the same class, resulting in heterogeneous ARV exposure (18). Inadequate ARV penetration may result in subtherapeutic concentrations within MGT organs and semen, thus creating the ideal environment for viral persistence and the development of drug-resistant viral strains (19, 20). Therefore, understanding ARV penetration into the MGT and its relationship with localized inflammation will be essential for elucidating the mechanisms underlying viral persistence in the MGT.

This narrative review describes the anatomical and physiological barriers throughout the MGT that contribute to its unique ARV pharmacology. We synthesize published data on the penetration of contemporary ARVs used for HIV treatment and prevention. Additionally, we identify important scientific gaps and future research directions related to ARV pharmacology in the MGT, including data surrounding novel ARV modalities and multidisciplinary computational approaches.

## LITERATURE SEARCH STRATEGY

A literature search was performed in PubMed for publications between May 1988 and October 2025. The final search was performed in January 2026. We used the following terms for our search strategy: “Semen,” “Seminal Plasma,” “Seminal Fluid,” “Seminal Vesicles,” “Prostate,” “Testes,” “Bulbourethral Glands,” “Epididymis,” “Vas Deferens,” in combination with “HIV,” “Physiology,” “Barrier,” “Drug Transporters,” and “Metabolizing Enzymes.” Additionally, we searched specific ARVs (“Abacavir,” “Emtricitabine,” “Lamivudine,” “Tenofovir Alafenamide,” “Tenofovir Disoproxil Fumarate,” “Zidovudine,” “Doravirine,” “Efavirenz,” “Rilpivirine,” “Bictegravir,” “Dolutegravir,” “Raltegravir,” “Atazanavir,” “Darunavir,” “Maraviroc”) in combination with “Male Genital Tract,” “Semen,” “Seminal Plasma,” and “Testes.” Finally, we used the following terms: “Broadly Neutralizing Antibodies,” “bNAbs,” “Cabotegravir/Rilpivirine,” “EFdA,” “Islatravir,” “Lenacapavir,” “Lipidomics,” “Microbiomics,” “Population Pharmacokinetics,” “PopPK,” “Physiologically-Based Pharmacokinetics,” “PBPK,” and “Proteomics” in combination with “HIV,” “Antiretrovirals,” “Semen,” and “Male Genital Tract.” Relevant peer-reviewed research articles, review articles, and case reports were included in this review.

## ANATOMY, PHYSIOLOGY, AND PHARMACOLOGICAL CONSIDERATIONS OF THE MGT

### Semen

**Fig 1:**
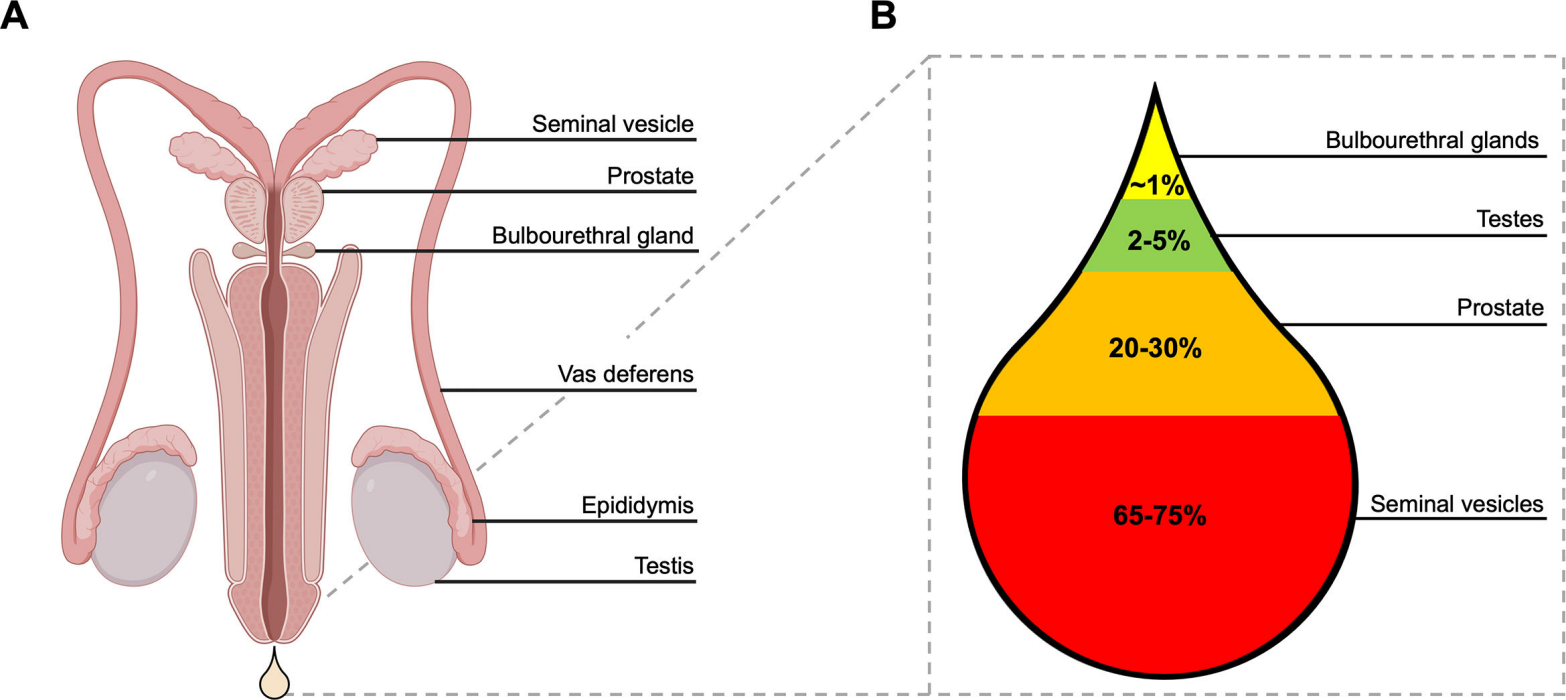
Anatomy of male genital tract organs (**A**) and relative contributions to total ejaculate by secretory male genital tract organs (**B**). Figure generated using data reported by Cao and Hendrix (23). Created in https://BioRender.com

ARV pharmacology throughout the MGT is most accurately characterized via tissue biopsy samples, but routine sampling is patently infeasible. Therefore, semen is used as a surrogate for understanding ARV pharmacology within the MGT (21). ARVs are quantified in SP from whole semen samples, providing an understanding of penetration into the MGT as a single compartment (21). Methods have been developed to split ejaculate into fractions according to the MGT organs from which ejaculate fractions originate (22). The MGT organs contributing to semen are the seminal vesicles, prostate, testes, and urethral/bulbourethral glands (23). The anatomical locations of these organs and their relative contributions to semen are displayed in Fig. 1A and B, respectively, and are discussed below (23).

Semen is a complex fluid mixture containing cells, proteins, lipids, and carbohydrates (23). Components of semen nourish, protect, and mobilize spermatozoa upon ejaculation (24). A single ejaculate is approximately 2–3 mL with a pH of 7.2–7.8 (25).

MGT organs contribute various molecules and secrete different volumes of fluid to the composition of semen (22). Accordingly, each MGT organ has unique physiology and pharmacology (22). MGT organs differ in drug metabolizing enzymes (DMEs) and drug transporter expression, epithelial barrier composition, and cellular composition, resulting in heterogeneous ARV penetration in these organs (22). Appreciating the pharmacological considerations of individual MGT organs and their relative contributions to semen provides valuable insight into the mechanisms driving heterogeneous ARV penetration.

### Seminal vesicles

The seminal vesicles are accessory glandular structures that secrete a thick fluid that constitutes 50%–80% of total ejaculate volume (26, 27). The seminal vesicle fluid provides nutrients for spermatozoa viability, promotes spermatozoa mobility, and suppresses immune activity to allow the spermatozoa to traverse the cervix, uterus, and fallopian tubes (26).

Immune cells in seminal vesicles, primarily CD4^+^ T cells, CD3^+^ T cells, and CD163^+^ macrophages, are effectively infected by HIV and may act as cellular reservoirs (28). In a study of recently deceased PWH, Deleage et al. noted that 5/7 autopsied seminal vesicles from men on ART contained HIV RNA and viral proteins, despite undetectable viral loads in the BP at the time of the most recent clinic visit (28). As seminal vesicles primarily contribute to the ejaculate and harbor HIV despite suppressive ART, studying ARV penetration into seminal vesicles is important for maintaining viral suppression in SP.

**Fig 2:**
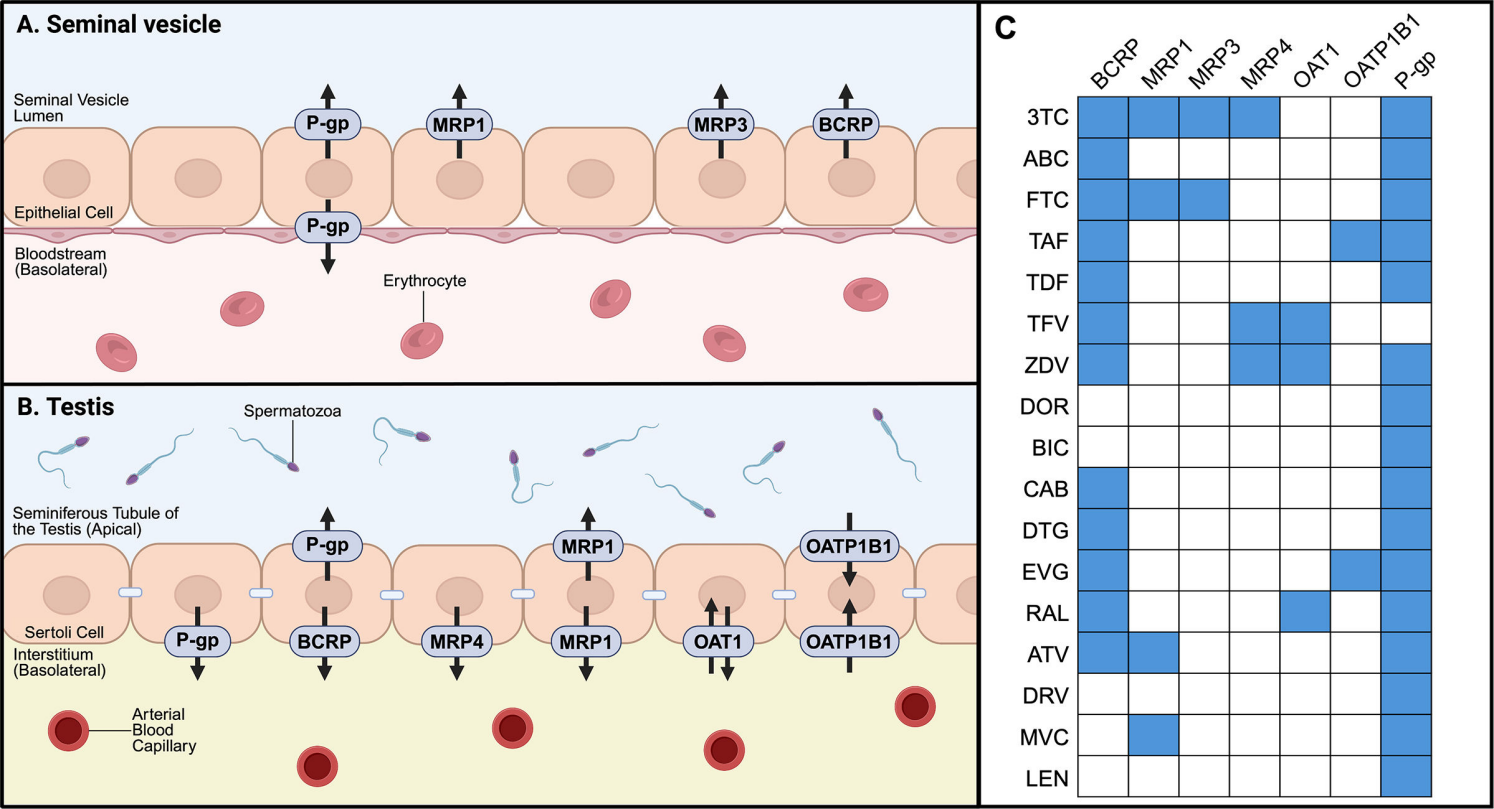
Localization of drug transporters on the seminal vesicle epithelium as confirmed by immunohistochemistry (**A**), and the blood-testes barrier as confirmed by confocal microscopy (**B**). **C** indicates the ARV substrates of the transporters described in **A** and **B**, denoted by blue squares. Abbreviations: 3TC, lamivudine; ABC, abacavir; ATV, atazanavir; BIC, bictegravir; BCRP, breast cancer resistance protein; CAB, cabotegravir; DOR, doravirine; DTG, dolutegravir; DRV, darunavir; EVG, elvitegravir; FTC, emtricitabine; LEN, lenacapavir; MRP, multidrug resistance protein; MVC, maraviroc; OAT, organic anion transporter; OATP, organic anion transporter protein; P-gp, P-glycoprotein; RAL, raltegravir; TAF, tenofovir alafenamide; TDF, tenofovir disoproxil fumarate; TFV, tenofovir; ZDV, zidovudine. Figure generated using data reported by Malinowski et al. (29) and Huang et al. (30). Panel C was created using data from references(31–44) and (45). Created in https://BioRender.com.

As shown in Fig. 2A, membrane transporters and solute carriers are present on the basolateral and apical membranes of the columnar cells forming the seminal vesicle epithelium (29). The seminal vesicle epithelium expresses mRNA of 11 ATP-binding cassette (ABC)-family transporters and 25 solute-linked carrier (SLC)-family transporters and carriers (29). ABC-family transporters function as efflux transporters via passive diffusion, co-transport, or counter-transport of endogenous molecules and xenobiotics (29). The ABC-family transporters multidrug resistance protein (MRP)1, MRP3, P-glycoprotein (P-gp), and breast cancer resistance protein (BCRP) are highly expressed on the apical membranes, likely for the secretion of molecules into SP (29). P-gp is also highly expressed on the basolateral membranes of the seminal vesicle epithelium to protect SP from toxins and xenobiotics (29).

SLC-family transporters within the seminal vesicles likely mediate the transport of ions and nutrients necessary for the production of seminal vesicle fluid and are expressed on both the basolateral and apical membranes of the seminal vesicle epithelium (29). SLC-family transporters support the secretory role of seminal vesicles, whereas the ABC-family transporters on the basolateral membrane suggest an additional protective role, limiting the penetration of xenobiotics (e.g., ARVs) (29).

### Prostate

The prostate is a chestnut-sized organ where the ejaculatory duct meets the urethra (46). The prostate provides 20%–40% of total ejaculate volume (23) and produces proteins essential for semen gelation, coagulation, and liquefaction (46).

HIV Gag protein has been found in the prostates of men with AIDS during autopsy, confirming that HIV resides in the prostate (47). Additionally, Smith et al. quantified HIV-1 RNA in the SP of nine men before and after prostatic massage (48). Prior to prostatic massage, 67% of participants had undetectable SP viral loads in multiple samples; following prostatic massage, all participants exhibited at least one instance of detectable SP viral load (48). Increased SP viral load following prostatic massage provides strong evidence that HIV localizes in the prostate and that the prostate can contribute to SP viral load (48).

The prostate contains DMEs and transporters that inhibit ARV penetration. Phase I DMEs in the prostate include multiple CYP1A, CYP2C, CYP3A isoforms as well as CYP1B1, CYP2D6, and CYP4B1 (49). Phase II enzymes relevant to ARV metabolism are present in the prostate as well, including glutathione S-transferase (GST) and UDP-glucuronosyltransferase (UGT) families (49). The majority of drug transporters in the prostate are efflux transporters from the ABC-transporter family (49), although mRNA expression of the SLC22A3 uptake transporter has been noted (49). DMEs and drug transporters in the prostate may prevent the accumulation of ARVs by metabolizing them directly in the prostate tissue and/or actively effluxing ARVs out of the prostate and back into systemic circulation (50).

### Testes

The testes produce spermatozoa and hormones (androgens) vital for sexual maturation and reproduction (51). Protecting haploid germ cells, the blood-testis barrier (BTB) separates testicular blood vessels and the seminiferous tubules (52). The BTB is comprised of Sertoli cells held together by tight junctions (52). These tight junctions significantly reduce ARV penetration into the testes (53, 54). Additionally, the BTB produces immunosuppressive factors (e.g., Fas ligand, programmed death-ligand 1, and indoleamine 2,3-dioxygenase) to limit immune cell recruitment and protect spermatozoa in the testes (55).

Given the unique immune environment and anatomical protection from xenobiotics, the testes are a prominent site for HIV persistence and compartmentalization in ART-suppressed PWH (56). T lymphocytes and testicular macrophages in the interstitial space between seminiferous tubules are the primary infected cells in the testes (57, 58). Although one study found that HIV can bind to the surface of germ cells, it is unlikely that HIV productively infects these cells (59). Interestingly, testicular Leydig cells, which produce testosterone, do not become infected with HIV-1 but can be infected by some strains of HIV-2 and simian immunodeficiency virus (SIV) (59).

As shown in Fig. 2B, the BTB contains several transporters relevant to ARVs, including ABC-family efflux transporters and SLC-family transporters (30). ABC-family transporters are localized on the basolateral and apical membranes of Sertoli cells, including BCRP, MRPs, and P-gp (60). SLC-family transporters include organic anion transporter polypeptides (OATPs), organic anion transporters (OATs), organic cation transporters (OCTs), concentrative nucleoside transporters (CNTs), and equilibrative nucleoside transporters (ENTs) (30, 61). Similarly, these are found on the apical and basolateral membranes of Sertoli cells (30, 60). A subset of ARVs that are substrates of these SLC transporters (e.g., tenofovir [TFV]) are likely to preferentially distribute into semen (31). Figure 2C lists ABC- and SLC-family transporters that have been imaged on the BTB via confocal microscopy and seminal vesicles via immunohistochemistry, cross-referenced with the ARV substrates discussed herein (32–44). In addition to drug transporters and carriers, CYP3A4 and CYP2D6 are found throughout the testicular tissue and may contribute to local drug metabolism, further reducing penetration of ARVs that are substrates of these DMEs (30).

### Bulbourethral glands

The bulbourethral glands secrete pre-ejaculatory fluid immediately prior to ejaculation (62). Politch et al. quantified HIV-1 RNA in the pre-ejaculate of 52 sexually active men with HIV on suppressive ART (63). Zero participants had detectable viral load in pre-ejaculate fluid, yet 10 participants had detectable viral loads in the SP (63). Therefore, the bulbourethral glands may not significantly contribute to the viral load observed in SP. To date, there are no available data on DMEs or drug transporters within the bulbourethral glands.

### Epididymides

The epididymides, connected to the testes, are tightly coiled tubes where spermatozoa mature and reside until ejaculation (64). The epididymides contract at ejaculation, propelling spermatozoa into the vas deferens (64).

Studies in cynomolgus macaques have demonstrated that epididymal T lymphocytes and macrophages are effectively infected by SIVmac251, an animal virus that mimics HIV by infecting primarily CD4^+^ and CCR5^+^ cells (65). These results suggest HIV may similarly infect these cells in humans. In semen samples from PWH, Muciaccia et al. found that spermatozoa with abnormal morphologies contained HIV DNA, but spermatozoa with normal morphologies did not (66). The mechanism of infection remains unclear, but authors hypothesized that infection occurred in the epididymis, where spermatozoa demonstrate non-specific uptake of RNA and DNA (66). Non-specific uptake of HIV RNA may result in the productive infection witnessed in spermatozoa with abnormal morphologies (66).

Similar to the testes, several influx, efflux, and bidirectional transporters are expressed within the epithelium of the epididymides to modulate xenobiotic exposure (67). Influx transporters expressed in the epididymis include ENT1, ENT2, OAT3, OATP1A4, OCTN2, and OCTN3, and efflux transporters include P-gp, MRP1, MRP5, MRP6, and MRP7 (67). As many ARVs are substrates of these transporters, efflux transporters likely prevent ARV accumulation in the epididymides.

### Vas deferens

The vas deferens is a duct that transports mature spermatozoa from the epididymides to the urethra during ejaculation via contraction of the surrounding smooth muscle (68). Anderson et al. demonstrated that vasectomized men (i.e., cut and sealed vas deferens) still had detectable SP viral loads (69). These data suggest HIV in semen comes primarily from accessory gland secretions and urethral epithelium rather than the testes and epididymides; vasectomies may not be a viable method to reduce HIV transmission (70). ARVs may readily penetrate the vas deferens due to expression of influx transporters, including OCT1, OATP1A4, and MRP2 (67).

## ARV EXPOSURE IN THE MGT

ARV distribution into the MGT varies between and within drug classes due to several factors, including anatomy, physiology, and drug physicochemical properties. Understanding this heterogeneity is essential for maintaining suppressive concentrations in the MGT and developing targeted therapies toward an HIV cure. In this section, we describe the pharmacokinetics (PK) of contemporarily prescribed ARVs with a focus on SP concentration to BP concentration ratios (SP:BP). These ratios quantify the magnitude to which ARVs preferentially distribute into the SP relative to BP. Ratios greater than 1 indicate preferential distribution into SP compared to BP, whereas ratios less than 1 indicate nonpreferential distribution into SP. If available, we also provide the area under the concentration-time curve ratios (SP:BP AUC). Similarly, SP:BP AUCs greater than 1 suggest greater drug exposure over the dosing interval in SP compared to BP.

### Nucleoside reverse transcriptase inhibitors

Nucleoside reverse transcriptase inhibitors (NRTIs) are part of first-line treatment regimens (71). NRTIs are intracellularly phosphorylated to their active metabolites (72). Here, we review NRTI pharmacology in the MGT. Detailed analyses of PK parameters organized by study are shown in Table 1.

**TABLE 1:** Studies evaluating NRTI penetration into the male genital tract^*a*^^,^^*b*^

Reference	Sample size	Cohort	ARV regimen	Dosing	Blood plasma concentration (ng/mL)	MGT matrix	MGT matrix concentration (ng/mL)	MGT matrix-to-BP ratios
Huang et al. (30)**¶**	13	Mixed Cohort	3TC, ATV, DRV, EFV, FTC, RTV, or TDF	Varied	3TC: 136 FTC: 214 TFV: 56	Testicular Tissue	3TC: 140 FTC: 251 TFV: 44	3TC: 0.4 FTC: 1.2 TFV: 0.8
Lorello et al. (73)**¶**	33	PWH	3TC, ABC, ATV, EFV, LPV/r, or ZDV	Varied	ABC: 40 (30–40) 3TC: 110 (90–130) ZDV: 2 (1–3)	SP	ABC: 218 (140–280) 3TC: 4,000 (3,000–5,000) ZDV: 110 (60–150)	ABC: 5.8 (5.0–6.8) 3TC: 40 (36–44) ZDV: Undefined
Van Praag et al. (74)	12	PWH	3TC, ABC, d4T, ZDV, and PIs	300 mg BID	ABC peak: 1,223 (925–2,051) ABC trough: 48–354	SP	ABC: 141–1,819	N/A^*c*^
Patterson et al. (75)†	8	PWOH	TDF/FTC	200 mg/300 mg	FTC C_24 h_: 47 (40–57) TFV C_24 h_: 41 (34–47)	SP	FTC C_24 h_: 253 (148–352) TFV C_24 h_: 23 (16–76)	FTC: 4.5 (3.3–6.1) TFV: 1.0 (0.6–1.4)
Valade et al. (76)**¶**	122	PWH	TDF/FTC ± PI/r ± RAL	200 mg QD	FTC C_24 h_: 136 (57–459) FTC AUC_0–24 h_: 12,000 (8,000–25,000)	SP	FTC C_24 h_: 850 (258–3,687) FTC AUC_0–24 h_: 38,040 (13,400–148,180)	FTC C_24 h_: 6.2 (4.5–8) FTC AUC_0–24 h_: 2.91 (0.84–10)
Haaland et al. (77)†	35	PWOH	EVG/c /TAF/FTC + DRV	Single oral dose	FTC: 1,268 (1,050–1,531)	Urethral swab Glans swab	FTC urethral: 36 (3–307) ng/swab FTC glans: 14 (<LLOQ-328) ng/swab TAF: undetected	N/A
Pereira et al. (78)†	9	PWH	3TC IDV ZDV	150 mg BID 800 mg TID 300 mg BID or 200 mg TID	3TC: 391 (175–793) ZDV: 64 (48–206)	SP	3TC: 2,701 (1,460–4,320) ZDV: 292 (194–438)	3TC: 9.1 (2.3–16) ZDV: 5.9 (0.95–14)
Taylor et al. (79)	12	PWH	NVP/3TC NVP/d4T	150 mg/200 mg BID 40 mg/200 mg BID	N/A	SP	N/A	3TC: 4.2–22
Charpentier et al. (80)†	18	PWH	DTG + 3TC	50 mg + 300 mg QD	3TC C_24 h_: 56 (21–180)	SP	3TC C_24 h_: 1,111 (67–4,352)	3TC C_24 h_: 24 (0.9–84)
Pereira et al. (81)	37	PWH	APV/3TC/ZDV APV	1,200 mg/150 mg/ 300 mg BID 1,200 mg BID	3TC 0–2 h: 880 3TC 8–12 h: 168 ZDV 0–2 h: 476 ZDV 8–12 h: 5	SP	3TC 0–2 h: 2,305 3TC 8–12 h: 2,497 ZDV 0–2 h: 459 ZDV 8–2 h: 127	3TC 0–2 h: 2.4 3TC 8–12 h: 15.7 ZDV 0–2 h: 2.2 ZDV 8–12 h: 33.8 3TC: 3.2 ZDV: 2.2
Dumond et al. (82)*	14	PWH	3TC/ZDV	150 mg/300 mg BID	3TC AUC_0–12 h_: 4,924 (4,373–6,237) h·ng/mL ZDV AUC_0–12 h_: 1,479 (1,175–1,748) h·ng/mL	SP	3TC AUC_0–12 h_: 31,084 (28,071–44,184) h·ng/mL ZDV AUC_0–12 h_: 3,790 (2,481–4,783) h·ng/mL	3TC AUC_0-12h_: 6.6 (4.1–9.1) ZDV AUC_0-12h_: 2.2 (1.4–2.9)
Imaz et al. (83)†	14	PWH	EVG/c /TAF/FTC EVG/c /TDF/FTC	150 mg/150 mg/ 10 mg/ 200 mg QD 150 mg/150 mg/ 300 mg/200 mg QD	TFV (TAF) C_24 h_: 9.1 (4.6–14.9) TFV (TDF) C_24 h_: 76 (43–378)	SP	TFV (TAF) C_24 h_: 110 (73–336) TFV (TDF) C_24 h_: 540 (236–6,980)	TFV (TAF) C_24h_ SP:BP: 12 TFV (TDF) C_24h_ SP:BP: 5.3
Lowe et al. (84)	30	PWH	ddI + TDF	Varied	TFV: 120,000	SP	TFV: 250,000	TFV: 3.3 (0.9–49)
Vourvahis et al. (85)	9	PWH	TDF	300 mg QD	N/A	SP	N/A	TFV C_24 h_ Mean: 5.1 ± 6.8
Henry et al. (86)†	6	PWH	ZDV	200 mg every 4–6 h	ZDV 0.75–1.25 h: 219 (141–342) ZDV 3.0–4.5 h: 101 (64–157)	SP	ZDV 0.75–1.25 h: 1,365 (1,215–1,531) ZDV 3.0–4.5 h: 879 (700–1,101)	ZDV 0.75–1.25 h: 6.2 (3.5–11) ZDV 3.0–4.5 h: 8.6 (4.4–17)
Cao et al. (87)**¶**	18	Mixed cohort	EFV ZDV IDV	100 mg every 4 h 10 mg/h IV infusion 200 mg every 2 h	ZDV: 181 (173–189)	SP	ZDV: 538 (456–635)	ZDV: 2.9 (2.4–3.6)
Dumond et al. (88)*	24	Mixed cohort	TAF/FTC TDF/FTC	Varied	AUC (h·ng/mL) TAF/FTC HIV (+): FTC: 10,441 (9,425–12,787) TFV: 318 (293–353) TDF/FTC HIV (−): FTC: 9,103 (6,416–10,745) TFV: 2,797 (1,579–3,558) TDF/FTC HIV (+): FTC: 7,520 (6,928–8,468) TFV: 2,635 (2,133–3,149)	SP	AUC (h·ng/mL) TAF/FTC HIV (+): FTC: 39,997 (26,526–50,939) TFV: 2,384 (1,791–4,318) TDF/FTC HIV (−): FTC: 23,413 (17,409–34,531) TFV: 2,702 (1,803–5,320) TDF/FTC HIV (+): FTC: 3,208 (25,706–36,263) TFV: 4,124 (2,359–9,044)	SP:BP (AUC) FTC/TAF HIV (+): TFV: 7.4 (5.2–12) FTC: 3.4 (2.4–5.3) FTC/TDF HIV (−): TFV: 1.1 (0.7–2.4) FTC: 2.8 (2.6–3.1) FTC/TDF HIV (+): TFV: 1.5 (0.8–3.6) FTC: 3.9 (3.6–4.6)
Ghosn et al. (89)**¶**	13	PWH	APV/r, EFV, LPV/r, IDV, SQV/r, T20, or TDF	300 mg QD	TDF: 52	SP	TDF: 101	TDF: 1.9
Seifert et al. (90)†	27	Mixed cohort	TDF/FTC TDF/FTC + EFV	300 mg/200 mg QD 300 mg/200 mg + 600 mg QD	TFV: 100 (81–118) FTC: 401 (335–467)	SP	TFV: 279 (162–483) FTC: 647 (453–924)	N/A

^*a*^ Summary of studies that quantified male genital tract concentrations of contemporary NRTIs. Values are standardized to median, median (range) [†], or median (IQR)^ [*]^unless stated otherwise. Units are standardized to ng/mL unless stated otherwise. Pilcrow (¶) indicates values that were calculated from raw data and are standardized to median (IQR)^.^

^*b*^ 3TC, lamivudine; ABC, abacavir; APV, amprenavir; ARV, antiretroviral; ATV, atazanavir; AUC, area under the curve; BID, twice daily; BP, blood plasma; C_12h_, 12-h trough concentration; C_24 h_, 24-h trough concentration; /c, cobicistat as pharmacoenhancer; d4T, stavudine; ddI, didanosine; DRV, darunavir; DTG, dolutegravir; EFV, efavirenz; EVG, elvitegravir; FTC, emtricitabine; IDV, indinavir; LLOQ, lower limit of quantification; LPV, lopinavir; MGT, male genital tract; MVC, maraviroc; NVP, nevirapine; PI, protease inhibitor; PWH, people with HIV; PWOH, people without HIV; QD, once a day; /r, ritonavir as pharmacoenhancer; RAL, raltegravir; RPV, rilpivirine; RTV, ritonavir; SP, seminal plasma; SQV, saquinavir; T20, enfuvirtide; TAF, tenofovir alafenamide; TDF, tenofovir disoproxil fumarate; TFV, tenofovir; Wk, week; ZDV, zidovudine.

^*c*^ "N/A" indicates not reported in associated reference.

#### Abacavir

Abacavir (ABC) demonstrates a mean SP:BP of 5.8 (73). Additionally, ABC rapidly penetrates the SP, achieving concentrations 10- to 20-fold higher than the *in vitro* concentration required to inhibit 50% of viral replication (IC_50_) for maximally susceptible HIV strains (70 ng/mL) within 2 h post-dose (74, 91). However, van Praag et al. showed that ABC concentrations in SP demonstrate high variability and rapid clearance, as a majority of participants (13/22) had undetectable SP ABC concentrations by the end of the 12-h dosing interval (74).

#### Emtricitabine

Following a single dose of TFV alafenamide/emtricitabine (TAF/FTC) in men without HIV, the FTC SP:BP AUC_0–14 d_ was 4.5 (75). In men with HIV, FTC SP:BP AUC_0–24 h_ was 2.9 (76). HIV RNA was detectable in the SP of 13.8% of men, although median SP AUC_0–24 h_ was not significantly different between men with detectable versus undetectable SP viral loads (76). Interestingly, FTC terminal half-lives were similar between BP (49 h) and SP (47 h) (75).

In a study examining FTC concentrations in swabs, FTC was detectable in 74% of urethral swabs and 67% of glans swabs of men without HIV (77). Urethral concentrations were estimated to be 4- to 8-fold higher than those in BP (77). Interestingly, glans samples gathered from uncircumcised participants revealed greater FTC concentrations than those measured in circumcised participants (77). In testicular tissue, Huang et al. reported FTC concentrations 1.8-fold higher than BP, aligning with trends observed in SP and urethral swabs (30).

#### Lamivudine

Lamivudine (3TC) SP:BP in men with HIV varies from 2.4 to 40 throughout the dosing interval (73, 78–81, 92). In a study of men with HIV, lower SP:BPs were observed early in the dosing interval (0–2 h; ratio 2.4) compared to the elimination phase (8–12 h; 15.7) (81). These results are likely driven by greater BP concentrations in the absorption phase, as SP concentrations during both phases were not significantly different (81). Supporting previous findings, a study in 14 men with HIV reported a median 3TC SP:BP AUC_0–12 h_ of 6.7 in men with HIV, and a 3TC half-life in SP twofold greater than that of BP (6.9 versus 2.7 h, respectively) (82).

Pereira et al. noted that SP HIV viral load decreased rapidly following 3TC-containing ART initiation, falling from 3.9 log_10_ copies/mL to undetectable in all participants within 14 days of treatment (78). Despite preferential distribution into the SP, a separate study using an ultrasensitive plasma viral load test (limit of detection 0.5 copies/mL) detected HIV RNA in 51% of SP samples at 48 weeks after initiating dolutegravir (DTG) + 3 TC (80). Interestingly, Huang et al. reported testicular tissue 3TC concentrations 52% lower than those in BP, suggesting 3TC does not uniformly preferentially penetrate into the MGT (30).

#### Tenofovir following TAF

A study in virally suppressed men switching from TFV disoproxil fumarate (TDF)-based ART to TAF-based ART reported a TFV SP:BP of 11.9 following TAF initiation (83). TFV trough concentrations in SP were 9.6-fold higher than the IC_50_ (11.5 ng/mL) (83, 93). All participants remained virally suppressed at 12 weeks post-switch (83). In ART-naïve healthy men, Haaland et al. detected TFV in <20% of urethral and glans swabs sampled up to 24 h after a single administration of oral TAF (77).

#### Tenofovir following TDF

Following TDF, median TFV SP:BP ranges from 3.3 to 5.4 in men with HIV (83–85). Patterson et al. noted that in men without HIV, TFV SP:BP AUC_1–14d_ was 1.0 (75). Importantly, studies have demonstrated that viral suppression in SP was achieved 14 days following TDF initiation or maintained following a switch to TDF (83, 85). Taken together, TFV preferentially distributes into the SP irrespective of prodrug formulation.

#### Zidovudine

Studies evaluating zidovudine (ZDV) have demonstrated SP:BPs >1 in men with HIV (78, 81, 86, 87). Samples collected at 0.75–1.25 h (time of maximum concentrations in BP) demonstrated a median SP:BP of 2.6 (86). In the same study, samples collected during the elimination phase of ZDV (3.0–4.5 h post-dose) demonstrated a higher median SP:BP of 10.6 (86). In alignment with individual timepoints, ZDV SP:BP AUC_0–12 h_ was 2.3 (82). ZDV’s half-life in SP (6.9 h) was 2.5-fold higher than that in BP (2.7 h), as similarly observed with 3TC (82). HIV RNA was undetectable in SP, suggesting effective viral suppression (81, 82).

### NRTI intracellular phosphorylated metabolites

Similar to SP:BP, the seminal mononuclear cell-to-peripheral blood mononuclear cell ratio (SMC:PBMC) describes the NRTI intracellular phosphorylated metabolite concentration in SMCs relative to PBMCs (72, 88, 94). The SMC:PBMC AUC describes the exposure of intracellular phosphorylated NRTIs in SMCs relative to PBMCs. These are pharmacologically active metabolites, and understanding their concentrations is essential for determining true efficacy (72, 82, 94). Additionally, the intracellular metabolite to endogenous nucleotide ratio (MER) is a measure of competitiveness between the NRTI metabolite and the respective endogenous nucleotides for virological activity (88) and is occasionally reported in the SP. The pharmacology of four NRTI intracellular metabolites in the MGT is discussed below and presented in Table 2.

**TABLE 2:** Studies evaluating NRTI intracellular phosphorylated metabolite penetration into the male genital tract^*a*^^,^^*b*^

Reference	Sample size	Cohort	ARV regimen	Dosing	PBMC concentration (fmol/10⁶ cells)	MGT matrix	MGT matrix concentration (fmol/10⁶ cells)	MGT matrix-to-PBMC ratios
Huang et al. (30)**¶**	13	Mixed cohort	3TC, ATV, DRV, EFV, FTC, RTV, or TDF	Varied	N/A^*c*^	Testicular tissue	3TCtp: 174 ng/mL FTCtp: 691 ng/mL TFVdp: 9 ng/mL	N/A
Imaz et al. (83)†	14	PWH	EVG/c/TAF/FTC EVG/c/TDF/FTC	150 mg/150 mg/10 mg/ 200 mg QD 150 mg/150 mg/300 mg/200 mg QD	TFVdp (TAF): 637 (213–1,154) TFVdp (TDF): 109 (20–361)	SMCs	TFVdp (TAF): 27 (10–468) TFVdp (TDF): 29 (3–421)	TFVdp (TAF) SMC:PBMC Mean: 0.06 (0.01–0.41) TFVdp (TDF) SMC:PBMC Mean: 0.28 (0.04–5.5)
Vourvahis et al. (85)	9	PWH	TDF	300 mg QD	TFVdp Mean: 118 ± 59	SMCs	TFVdp Mean: 1,544 ± 2,079	TFVdp C_24h_ Mean: 12
Dumond et al. (82)*	14	PWH	3TC/ZDV	150 mg/ 300 mg BID	3TCtp AUC_0–12 h_: 108,600 (78,687–164,984) h·fmol/10⁶ cells ZDVtp AUC_0–12 h_: 1,460 (1,241–2,172) h·fmol/10⁶ cells	SMCs	3TCtp AUC_0–12 h_: 82,068 (64,342–139,404) h·fmol/10⁶ cells ZDVtp AUC_0–12 h_: 646 (361–1,029) h·fmol/10⁶ cells	3TCtp AUC_0–12 h_: 1.0 (0.62–1.3) ZDVtp AUC_0–12 h_: 0.36 (0.30–0.37)
Dumond et al. (88)*	24	Mixed cohort	TAF/FTC TDF/FTC	Varied	TAF/FTC PWH: FTCtp: 7,756 (7,161–14,530) TFVdp: 935 (684–2,024) TDF/FTC PWOH: FTCtp: 6,460 (5,060–7,837) TFVdp: 116 (108–164) TDF/FTC PWH: FTCtp: 6,397 (5,263–9,408) TFVdp: 192 (156–237)	SMCs	TAF/FTC HIV (+): FTCtp: 179 (135–356) TFVdp: 35 (24–78) TDF/FTC HIV (−): FTCtp: 88 (62–156) TFVdp: 21 (4–39)TDF/FTC HIV (+): FTCtp: 83 (52–137) TFVdp: 21 (6–58)	TAF/FTC PWH:FTCtp: 0.01 (0.01−0.03) TFVdp: 0.03 (0.01−0.07) TDF/FTC PWOH: FTCtp: 0.02 (0.01–0.03) TFVdp: 0.22 (0.04–0.32) TDF/FTC PWH): FTCtp: 0.01 (0.01–0.02) TFVdp: 0.09 (0.04–0.39)
Seifert et al. (90)†	27	Mixed cohort	TDF/FTC TDF/FTC + EFV	300 mg/200 mg 300 mg/200 mg + 600 mg`	TFVdp: 85 (73–98) FTCtp: 5.4 (5–6)	SMCs	TFVdp: 22 (6–79) FTCtp: 300 (200–500)	N/A

^*a*^ Summary of studies that quantified male genital tract concentrations of intracellular phosphorylated metabolites of contemporary NRTIs. Values are standardized to median only, median (range) [†], or median (IQR) [*] unless stated otherwise. Units are standardized to fmol/10^6^ cells unless stated otherwise. Pilcrow (¶) indicates values that were calculated from raw data and are standardized to median (IQR)..

^*b*^ 3TC, lamivudine; 3TCtp, lamivudine triphosphate; ARV, antiretroviral; ATV, atazanavir; AUC, area under the curve; BID, twice daily; C_24 h_, 24-h trough concentration; /c, cobicistat as pharmacoenhancer; DRV, darunavir; EFV, efavirenz; EVG, elvitegravir; FTC, emtricitabine; FTCtp, emtricitabine triphosphate; MGT, male genital tract; PBMC, peripheral blood mononuclear cell; PI, protease inhibitor; PWH, people with HIV; PWOH, people without HIV; QD, once a day; RTV, ritonavir; SMC, seminal mononuclear cell; TAF, tenofovir alafenamide; TDF, tenofovir disoproxil fumarate; TFV, tenofovir; TFVdp, tenofovir diphosphate; ZDV, zidovudine; ZDVtp, zidovudine triphosphate.

^*c*^ N/A, not reported in associated reference.

#### FTC triphosphate

Intracellular FTC triphosphate (FTCtp) concentrations in PBMCs and SMCs were evaluated across multiple studies; FTCtp concentrations were detectable in SMCs (88) and testicular tissue (30). FTCtp SMC:PBMC in men with and without HIV was <1, indicating lower FTC phosphorylation potential in the MGT compared to BP (88). Additionally, the median MER for FTCtp compared to deoxycytidine triphosphate in SMCs of men with HIV was >1.5 following co-administration with TAF or TDF, which exceeded the value necessary to achieve 90% effective concentration in CD4^+^ T cells (EC_90_; 0.07) (88, 95). In testicular tissue, Huang et al. reported an FTCtp concentration of 692 ng/mL (30).

#### 3TC Triphosphate

One group investigated 3TC triphosphate (3TCtp) in SMCs and PBMCs throughout the dosing interval, reporting an SMC:PBMC AUC_0–12 h_ of 1.0 (82). In testicular tissue, 3TCtp concentrations were detectable but varied widely among the three participants in the study (109–3,227 ng/mL) (30).

#### TFV diphosphate

Imaz et al. reported significantly higher TFV diphosphate (TFVdp) SMC:PBMC following TDF administration (0.28) compared to TAF (0.06) (83). Similarly, another group reported a TFVdp SMC:PBMC of 0.09 following TDF/FTC and 0.03 following TAF/FTC in men with HIV (88). Men without HIV receiving TDF/FTC had a twofold higher SMC:PBMC compared to men with HIV, although this was not statistically significant (88). Interestingly, MERs were approximately 1 following the administration of either TFV prodrug, indicating both formulations achieve sufficient intracellular concentrations in uninfected cells in the MGT (88, 90, 95).

#### ZDV triphosphate

In contrast to 3TCtp, ZDV triphosphate (ZDVtp) demonstrated a median SMC:PBMC AUC_0–12 h_ value of 0.36, suggesting lower exposure in SMCs compared to PBMCs throughout the dosing interval (82).

### Non-nucleoside reverse transcriptase inhibitors

Here, we discuss three contemporary non-nucleoside reverse transcriptase inhibitors (NNRTIs). Additional study details and summaries are shown in Table 3.

**TABLE 3:** Studies evaluating NNRTI penetration into the male genital tract^*a*^^,^^*b*^

Reference	Sample size	Cohort	ARV regimen	Dosing	Blood plasma concentration (ng/mL)	MGT matrix	MGT matrix concentration (ng/mL)	MGT matrix-to-BP ratios
Huang et al. (30)**¶**	13	Mixed cohort	3TC, ATV, DRV, EFV, FTC, RTV, or TDF	Varied	EFV: 7,448	Testicular tissue	EFV: 1,856	EFV: 0.25
Lorello et al. (73)**¶**	33	PWH	EFV, ABC, 3TC, ATV, LPV/r, or ZDV	Varied	EFV: 2,420 (2,050–2,790)	SP	EFV: 200 (150–260)	EFV: 0.09 (0.07–0.11)
Cao et al. (87)**¶**	18	Mixed cohort	EFV IDV ZDV	100 mg every 4 h 200 mg every 2 h 10 mg/h IV infusion	EFV: 2,964 (2,358–3,726)	SP	EFV: 196 (159–241)	EFV: 0.05
Scévola et al. (96)†	30	PWH	DOR + TAF/FTC	100 mg + 200/25 mg QD	Total DOR C_24 h_: 363 (77–566) Unbound DOR C_24 h_: 90 (37–172)	SP	Total DOR C_24 h_: 127 (31–272) Unbound DOR C_24 h_: 104 (27–218)	Total DOR C_24 h_: 0.35 (0.40–0.48) Unbound DOR C_24h_: 1.5 (0.83–2.8)
Ghosn et al. (89)**¶**	13	PWH	APV/r, EFV, IDV, LPV/r, SQV/r, T20, or TDF	600 mg QD	EFV: 1,330	SP	Undetectable	N/A^*c*^
Lowe et al. (97)†	12	PWH	3TC/ZDV + EFV ddI + d4T + NFV 3TC/ddI/r/SQV	150 mg/300 mg BID + 600 mg QD 400 mg QD + 40 mg +1,250 mg BID 300 mg/400 mg + 100 mg/1,600 mg QD	EFV: 1,300 (1,100–3,700)	SP	BLQ	N/A
Avery et al. (98)*	6	PWH	EFV	600 mg QD Switch to 100 mg every 4 hours	Total EFV: 1,940 (1,593–4,555) Unbound EFV: 4.2 (3.4–7.7)	SP	Total EFV: 136 (66–255) Unbound EFV: 5.4 (4.1–8.6)	Total EFV: 0.04 (0.03–0.05) Unbound EFV: 1.2 (0.99–1.4)
Mora-Peris et al. (99)**¶**	13	PWH	TDF/FTC + RPV	200 mg/245 mg + 25 mg	RPV: 55 (40–57)	SP	RPV: 4.9 (4.2–5.6)	RPV: 0.10 (0.08–0.11)
Gutierrez-Valencia et al. (100)	36	PWH	TDF/FTC + DRV/r TDF/FTC + EVG/c TDF/FTC + RPV	300 mg/200 mg + 800 mg/100 mg QD 300 mg/200 mg + 150 mg/150 mg QD 300 mg/200 mg + 25 mg QD	RPV mean (range): 105 (86–142)	SP	RPV mean (range): 24 (18–27)	RPV mean (range): 0.19 (0.15–0.31)
Lê et al. (101)**¶**	35	PWH	MVC + PI/r RAL RPV	150 mg BID or 300 mg BID ±400 mg BID ±25 mg QD	RPV C_24h_: 111 (66–163)	SP	N/A	RPV: 0.02 (0.01–0.05)
Chan et al. (102)**¶**	119	PWH	ATV/r or LPV/r EFV or NVP	ATV/r: 150 mg QD, 200 mg QD, 300 mg QD, or 400 mg QD EFV: 600 mg QD LPV/r: 3 × 133 mg BID NVP: 200 mg BID	EFV: 1,705 (1,615–2,648)	SP	Undetectable	N/A

^*a*^ Summary of studies that quantified male genital tract concentrations of contemporary NNRTIs. Values are standardized to median, median (range) [†], or median (IQR)^ [*]^unless stated otherwise. Units are standardized to ng/mL unless stated otherwise.Pilcrow (¶) indicates values that were calculated from raw data and are standardized to median (IQR).

^*b*^ 3TC, lamivudine; ABC, abacavir; APV, amprenavir; ARV, antiretroviral; ATV, atazanavir; BID, twice daily; BLQ, below limit of quantification; BP, blood plasma; /c, cobicistat as pharmacoenhancer; C_24 h_, 24-h trough concentration; d4T, stavudine; ddI, didanosine; DOR, doravarine; DRV, darunavir; EFV, efavirenz; EVG, elvitegravir; FTC, emtricitabine; IDV, indinavir; LPV, lopinavir; MGT, male genital tract; MVC, maraviroc; NFV, nelfinavir; NVP, nevirapine; PI, protease inhibitor; PWH, people with HIV; QD, once a day; /r, ritonavir as pharmacoenhancer; RAL, raltegravir; RPV, rilpivirine; RTV, ritonavir; SP, seminal plasma; SQV, saquinavir; T20, enfuvirtide; TAF, tenofovir alafenamide; TDF, tenofovir disoproxil fumarate; TFV, tenofovir; ZDV, zidovudine.

^*c*^ N/A, not reported in associated reference.

#### Doravirine

Scévola et al. studied doravirine (DOR) concentrations in men with HIV on stable ART who switched to a DOR + TAF/FTC regimen (96). Total and protein-unbound DOR concentrations in SP at the end of the dosing interval were measured (96). Eight weeks after switching, median total DOR concentrations were 127 ng/mL in SP and 363 ng/mL in BP (SP:BP: 0.35) (96). However, DOR protein-binding potential was lower in SP (12.8%) than that in BP (81.3%), resulting in higher SP:BPs when correcting for protein binding (1.5) (96). This indicates higher concentrations of pharmacologically active DOR in SP compared to BP (96). Importantly, 14/15 men had undetectable viral loads in SP (96).

#### Efavirenz

Efavirenz (EFV) concentrations have been reported as either undetectable or unquantifiable in two studies (89, 97). Cao et al. reported low but detectable EFV concentrations (196 ng/mL) in SP (87). Therein, the SP:BP was 0.05 (87), an estimate supported by other studies (0.04-0.1) (98, 103). The protein-binding potential of EFV in SP was 95.3% compared to 99.8% in BP (98). Interestingly, all participants in that study maintained undetectable SP viral loads despite non-preferential EFV penetration (97). In testicular tissue, Huang et al. noted a testicular tissue-to-BP ratio of 0.25 alongside undetectable viral loads (30).

#### Rilpivirine

Rilpivirine (RPV) poorly penetrates the MGT following oral administration, demonstrating SP:BPs of 0.19 (after switching from a nevirapine-based regimen) and 0.09 (treatment-naïve) at the end of the 24-h dosing interval (99, 100). The greater SP:BP may be a result of residual CYP3A4 induction from nevirapine exposure, resulting in lower BP concentrations compared to those in the treatment-naïve group (99, 100, 104). In a study of 12 men with HIV, mean total RPV concentrations in SP (24 ng/mL) exceeded the protein-binding-adjusted concentration required for 90% inhibition of viral replication (PA-IC_90_) for wild-type HIV-1 (12.1 ng/mL) (105), and all participants achieved undetectable SP viral loads by week 12 (100). The protein-binding potential of RPV in SP was reported to be 99.5% (101).

### Integrase strand transfer inhibitors

Integrase strand transfer inhibitors (INSTIs) are known for their high barrier to resistance and tolerability. They are recommended as first-line therapy for treatment-naïve PWH (106). Here, we discuss INSTI pharmacology in the MGT, with study details summarized and provided in Table 4.

**TABLE 4:** Studies evaluating INSTI penetration into the male genital tract^*a*^^,^^*b*^

Reference	Sample size	Cohort	ARV regimen	Dosing	Blood plasma concentration (ng/mL)	MGT matrix	MGT matrix concentration (ng/mL)	MGT matrix-to-BP ratios
Haaland et al. (77)†	35	PWOH	EVG/c /TAF/FTC + DRV	Single oral dose	N/A^*c*^	Urethral swab Glans swab	EVG urethral: <LLOQ EVG glans: <LLOQ	N/A
Charpentier et al. (80)†	18	PWH	DTG + 3TC	50 mg + 300 mg QD	Total DTG single dose C_24 h_: 1,900 (20–2,728) Total DTG Wk24 C_24 h_: 1,812 (670–2,466)	SP	DTG single dose C_24 h_: 206 (5–394) DTG Wk24 C_24 h_: 243 (54–1,928)	Total DTG C_24 h_ D0: 0.11 (0.02–0.24) Total DTG Wk24 C_24 h_: 0.14 (0.03–1.0)
Gutierrez-Valencia et al. (100)	36	PWH	TDF/FTC + DRV/r TDF/FTC + EVG/c TDF/FTC + RPV	300 mg/200 mg + 800 mg/100 mg QD 300 mg/200 mg + 150 mg/150 mg QD 300 mg/200 mg + 25 mg QD	EVG mean (range): 238 (109–571)	SP	EVG mean (range): 85 (44–206)	EVG mean (range): 0.43 (0.20–0.92)
Lê et al. (101)	35	PWH	MVC + PI/r RAL RPV	150 mg BID or 300 mg BID ± 400 mg BID ±25 mg QD	RAL C_12 h_: 63 (34–98) RAL Glucuronide C_12h_: 133 (59–330)	SP	N/A	RAL: 6.8 (3.3–14) RAL glucuronide: 16 (4.9–54)
Imaz et al. (107)†	15	PWH	BIC/TAF/FTC	50 mg/200 mg/25 mg QD	Total BIC C_24 h_: 2,640 (424–10,300) Unbound BIC: 5.2 (1.1–91)	SP	Total BIC C_24 h_: 65 (20–923) Unbound BIC: 31 (4.6–508)	BIC: 0.03 (0.01–0.31)
Greener et al. (108)†	12	PWOH	DTG	50 mg QD	DTG single dose C_24h_: 704 (580–874) DTG multiple dose C_24h_: 954 (554–1,093)	SP	DTG single dose C_24 h_: 46 DTG multiple dose C_24 h_: 57 (47–94)	DTG C_24 h_: 0.06 DTG single dose SP:BP AUC_0–24 h_: 0.07 DTG multiple dose SP:BP AUC_0–24 h_: 0.07
Imaz et al. (109)†	15	PWH	ABC/3TC + DTG	600 mg/300 mg + 50 mg QD	Total DTG Wk4: 1,200 (124–2,290) Total DTG Wk24: 1,420 (353–3,100) Unbound DTG Wk4: 5.2 (3.1–10) Unbound DTG Wk24: 6.2 (3.0–12)	SP	Total DTG Wk4: 92 (15–331) Total DTG Wk24: 129 (38–423) Unbound DTG Wk4: 34 (5–159) Unbound DTG Wk24: 56 (13–203)	Total DTG Wk 4: 0.08 (0.04–0.21) Total DTG Wk 24: 0.09
Imaz et al. (110)†	10	PWH	EVG/c /TDF/FTC	150 mg/150 mg/300 mg/200 mg	EVG: 277 (65–1,790)	SP	EVG: 169 (13–792)	EVG: 0.39 (0.20–0.92)
Calcagno et al. (111)†	8	PWOH	RAL	Days 1–4: 400 mg BID Day 5: 400 mg QD	RAL 2–4 h: 651 (438–1,020) RAL 11–12 h: 151 (51–443)	SP	RAL 2–4 h: 953 (541–1,706) RAL 11–12 h: 937 (212–3,582)	RAL 2–4 h: 1.6 (1.3–2.1) RAL 11–12 h: 6.4 (3.7–9.3)
Barau et al. (112)†	19	PWH	RAL	400 mg BID	RAL: 206 (106–986)	SP	RAL: 345 (83–707)	RAL: 1.4 (0.52–6.7)
Osborne et al. (113)†	38	PWH	MVC + RAL	Varied	RAL: 246 (40–1,380)	SP	RAL: 723 (282–1,890)	RAL: 4.9 (0.49–8.9)
Lê et al. (114)*	103	PWH	ETR RAL	200 mg/400 mg BID	Total RAL C_12 h_ Wk48: 267 (111–737) Unbound RAL C_12 h_ Wk48: 30 (13–83)	SP	Total RAL C_12 h_ Wk48: 560 (348–798) Unbound RAL C_12 h_ Wk48: 397 (217–512)	Total RAL C_12 h_ Wk48: 12 (6.5–18) Unbound RAL C_12 h_ Wk48: 5.7 (2.0–12)

^*a*^ Summary of studies that quantified male genital tract concentrations of contemporary INSTIs. Values are standardized to median, median (range) [†], or median (IQR)^ [*]^unless stated otherwise. Units are standardized to ng/mL unless stated otherwise. Pilcrow (¶) indicates values that were calculated from raw data and are standardized to median (IQR).

^*b*^ 3TC, lamivudine; ABC, abacavir; ARV, antiretroviral; AUC, area under the curve; BIC, bictegravir; BID, twice daily; BP, blood plasma; /c, cobicistat as pharmacoenhancer; C_12 h_, 12-h trough concentration; C_24 h_ , 24-h trough concentration; DTG, dolutegravir; ETR, etravirine; EVG, elvitegravir; FTC, emtricitabine; LLOQ, lower limit of quantification; MGT, male genital tract; MVC, maraviroc; PI, protease inhibitor; PWH, people with HIV; PWOH, people without HIV; QD, once a day; /r, ritonavir as pharmacoenhancer; RAL, raltegravir; RPV, rilpivirine; SP, seminal plasma; TAF, tenofovir alafenamide; Wk, Week.

^*c*^ N/A, not reported in associated reference.

#### Bictegravir

In a study of 15 ART-naïve PWH, the median total bictegravir (BIC) concentration was 65.5 ng/mL in SP and 2,640 ng/mL in BP (SP:BP: 0.02) (107). Despite non-preferential penetration, BIC demonstrated lower protein-binding potential in the SP (51.1%) compared to BP (99.8%); the median protein-unbound concentrations were 31.2 ng/mL and 5.2 ng/mL in SP and BP, respectively (107). When correcting for protein binding, the median SP:BP was 6.0 (107). Viral suppression in the SP occurred by day 28 following initiation in 14/15 participants (107).

#### Dolutegravir

Reported total DTG SP:BPs range from 0.07 to 0.12 at the end of the 24-h dosing interval (80, 108, 109). The reported protein-binding potential of DTG in SP was 52.0%, compared to 99.5% in BP (109). The estimated protein-unbound DTG concentrations in SP at steady state (56 ng/mL) were 214-fold above the *in vitro* IC_50_ (0.21 ng/mL) (115) and ninefold greater than those in BP (109). Interestingly, the median time to viral suppression was 4 weeks in SP versus 12 weeks in BP (109).

#### Elvitegravir

Cobicistat-boosted elvitegravir (EVG/c) SP:BPs were 0.39 in virally suppressed PWH and 0.43 in treatment-naïve PWH (100, 110). EVG trough concentrations in SP were 84.6 ng/mL (virally suppressed) and 169.5 ng/mL (treatment-naïve) (100, 110). By week 12 following initiation, all 12 treatment-naïve participants achieved undetectable SP viral loads (100). In both cohorts, EVG concentrations in SP achieved >20-fold the *in vitro* half-maximal effect concentration (EC_50_; 0.55 ng/mL) (100, 110, 116). Following a single dose, EVG was detected in <20% of urethral and glans swabs within 24 h (77).

#### Raltegravir

In contrast to the other INSTIs, raltegravir (RAL) consistently demonstrates SP:BPs ranging from 1.4 to 6.5, with higher SP:BP measured at the end of the dosing interval compared to 2–4 h post-dose (111–114). Following the trend of other ARVs, the protein-binding potential was lower in the SP (29%) compared to BP (88%); the median protein-unbound RAL concentrations were 397 ng/mL and 30 ng/mL for SP and BP, respectively (112). When correcting for protein binding, the median SP:BP ratio was 12.0 (112). In a study evaluating isolated HIV shedding in semen, those receiving RAL-containing regimens achieved undetectable viral loads by 2 weeks (113). Of note, isolated viral shedding was confirmed in 2/15 participants receiving RAL, which was significantly associated with lower RAL SP concentrations (113).

### Protease inhibitors

Protease inhibitors (PIs) are typically co-administered with ritonavir or cobicistat to harness PK-enhancing effects. Due to their high drug-drug interaction potential, PIs have become more limited in the treatment algorithms (117). Two contemporary PIs are discussed here, and the studies are summarized in Table 5.

**TABLE 5:** Studies evaluating PI penetration into the male genital tract^*a*^^,^^*b*^

Reference	Sample size	Cohort	ARV regimen	Dosing	Blood plasma concentration (ng/mL)	MGT matrix	MGT matrix concentration (ng/mL)	MGT matrix-to-BP ratios
Huang et al. (30)**¶**	13	Mixed cohort	3TC, ATV, DRV, EFV, FTC, RTV, or TDF	Varied	ATV: 11,300 DRV: 2,209	Testicular tissue	ATV: 1,029 DRV: 459	ATV: 0.79 DRV: 0.21
Lorello et al. (73)**¶**	33	PWH	3TC, ABC, ATV, EFV, LPV/r, or ZDV	Varied	ATV: 216 (8–420) ATV/r: 668 (2–1,330)	SP	ATV: 104 (20–180) ATV/r: 124 (40–200)	ATV: 0.59 (0.41–0.76) ATV/r: 0.34 (0.21–0.46)
Haaland et al. (77)†	35	PWOH	EVG/c/TAF/FTC + DRV	Single oral dose	DRV: 2,548 (1,914–3,392)	Urethral Glans	DRV urethral: 25 (2–52) ng/swab DRV Glans: 6 (<LLOQ-149) ng/swab	N/A^*c*^
Gutierrez-Valencia et al. (100)**¶**	36	PWH	TDF/FTC + DRV/r TDF/FTC + EVG/c TDF/FTC + RPV	300 mg/200 mg + 800 mg/100 mg QD 300 mg/200 mg + 150 mg/150 mg QD 300 mg/200 mg + 25 mg QD	DRV mean (range): 1,831 (1,223–1,831)	SP	DRV mean (range): 183 (128–226)	DRV mean (range): 0.10 (0.08–0.14)
Van Leeuwen et al. (118)*	15	PWH	ATV ± RTV	300 mg or 400 mg QD ± 100 mg QD	ATV: 2,100 (617–3,937) ¶	SP	ATV: 210 (105–315) ¶	ATV: 0.10 (0.08–0.17)
Chan et al. (102)**¶**	119	PWH	ATV/r or LPV/r EFV or NVP	ATV/r: 150 mg QD, 200 mg QD, 300 mg QD, or 400 mg QD EFV: 600 mg QD LPV/r: 3 × 133 mg BID NVP: 200 mg BID	ATV: 630 (195–1,860)	SP	ATV: 87 (21–230)	ATV: 0.14
Taylor et al. (103)†	18	PWH	DRV/r + NRTI	800 mg/100 mg QD	DRV 1–3 h: 5,579 (4,639–7,505) DRV 4–6 h: 3,734 (2,935–4,586) DRV 22–24 h: 2,445 (1,365–3,167)	SP	DRV 1–3 h: 588 (509–778) DRV 4–6 h: 490 (479–640) DRV 22–24 h: 217 (172–261)	DRV 1–3 h: 0.11 (0.09–0.15) DRV 4–6 h: 0.13 (0.07–0.18) DRV 22–24 h: 0.11 (0.09–0.15)
Arenas-Pinto et al. (119)*	67	PWH	ATV + OTT or DRV	Varied	N/A	SP	ATV: 129 (63–368) DRV: 660 (339–1,089)	N/A
Lambert-Niclot et al. (120)*	45	PWH	DRV/r ± NRTI	600 mg/100 mg BID	Total DRV C_12 h_: 3,200 Unbound DRV C_12 h_: 212	SP	DRV C_12 h_: 344 (149–652)	DRV C_12 h_: 0.08 (0.05–0.22)
Brown et al. (121)†	12	PWOH	DRV/r + ETR	Days 1–7: 600 mg/100 mg + 200 mg BID Day 8: 600 mg/100 mg + 200 mg QD	DRV single dose C_12 h_: 1,710 (880–3,820) DRV multiple dose C_12 h_: 2,800 (1,170–6,910)	SP	DRV single dose C_12h_: 152 DRV multiple dose C_12 h_: 267 (116–5,250)	DRV single dose: 0.08 DRV multiple dose: 0.15 (0.08–0.26)
Gay et al. (122)†	12	PWH	DRV/r + ETR	800 mg/100 mg QD + 400 mg QD or 200 mg BID	N/A	SP	DRV: (9–1,296)	DRV: 0.16 (0.002–0.19)

^*a*^ Summary of studies that quantified male genital tract concentrations of contemporary PIs. Values are standardized to median, median (range) [†], or median (IQR) ^ [*]^unless stated otherwise. Units are standardized to ng/mL unless stated otherwise. Pilcrow (¶) indicates values that were calculated from raw data and are standardized to median (IQR).

^*b*^ 3TC, lamivudine; ABC, abacavir; ARV, antiretroviral; ATV, atazanavir; AUC, area under the curve; MGT, male genital tract; BID, twice daily; BP, blood plasma; /c, cobicistat as pharmacoenhancer; C_12 h_, 12-h trough concentration; Cobi, cobicistat; DRV, darunavir; EFV, efavirenz; ETR, etravirine; EVG, elvitegravir; FTC, emtricitabine; LLOQ, lower limit of quantification; LPV, lopinavir; NRTI, nucleotide reverse transcriptase inhibitor; NVP, nevirapine; OTT, ongoing triple therapy; PI, protease inhibitor; PWH, people with HIV; PWOH, people without HIV; QD, once a day; /r, ritonavir as pharmacoenhancer; RTV, ritonavir; RPV, rilpivirine; SP, seminal plasma; TAF, tenofovir alafenamide; TDF, tenofovir disoproxil fumarate; TFV, tenofovir; ZDV, zidovudine.

^*c*^ N/A, not reported in associated reference.

#### Atazanavir

Studies investigating atazanavir (ATV) in the MGT report SP:BPs <1 (73, 102, 118). Van Leeuwen et al. reported a weak correlation between ATV BP and SP concentrations (Spearman’s rho = 0.46), and this finding was confirmed by Chan et al., reporting high interindividual SP:BP variability (range: 0.05–0.97) (102, 118). Three independent studies reported median ATV SP concentrations above the PA-IC_90_ (14 ng/mL) (73, 102, 118, 123). Lastly, ATV demonstrated testicular concentrations 79% those of BP, further confirming poor MGT penetration overall (30).

#### Darunavir

Similar to ATV, darunavir (DRV) poorly penetrates into the MGT, consistently demonstrating a SP:BP of 0.08–0.16 across multiple studies (18, 77, 100, 119–122, 124). Lambert-Niclot et al. reported a median total DRV SP concentration approximately sixfold higher than the DRV EC_50_ against wild-type HIV-1 (55 ng/mL) (120, 125). A single- and multiple-dose study in people without HIV revealed that the protein-binding potential of DRV in SP was 14.0% compared to approximately 95% in BP (121). In swabs of the inner urethra, Haaland et al. reported that DRV concentrations peaked at 4 h (23 ng/swab); DRV was still detectable on swabs of the glans of the penis after 24 h (77). In the same study, DRV concentrations were greater in the glans swabs from uncircumcised versus circumcised men, similar to what was observed with FTC (77). In testicular tissue, DRV is detectable, with a tissue penetration ratio of 0.21 (30). Arenas-Pinto et al. reported that 2/34 men on DRV monotherapy had detectable HIV RNA in semen, but viral load was below the lower limit of detection (119).

### Entry inhibitors

Maraviroc (MVC) is a C-C chemokine receptor type 5 inhibitor used for HIV treatment (126). MVC is a substrate of CYP3A4, and its exposure is significantly affected by co-administration of CYP inducers and inhibitors (127). Below is a summary of the PK of MVC in the MGT, and study details are provided in Table 6.

**TABLE 6:** Studies evaluating maraviroc penetration into the male genital tract^*a*^^,^^*b*^

Reference	Sample size	Cohort	ARV regimen	Dosing	Blood plasma concentration (ng/mL)	MGT matrix	MGT matrix concentration (ng/mL)	MGT matrix-to-BP ratios
Lê et al. (101)**¶**	35	PWH	MVC + PI/r RAL RPV	150 mg BID or 300 mg BID ± 400 mg BID ± 25 mg QD	MVC C_12 h_: 52 (33–83)	SP	N/A^*c*^	MVC: 1.5 (0.50–4.6)
Osborne et al. (113)†	38	PWH	RAL/MVC + background	Varied	MVC: 109 (44–377)	SP	MVC: 804 (153–4,920)	MVC: 9.7
Brown et al. (128)†	12	PWOH	MVC	300 mg BID for Days 1–7 300 mg Day 8	Single dose: MVC C_12 h_: 20 (7–51) MVC AUC_0–12 h_: 1,680 (510–3,015) h·ng/mL Multiple dose: MVC C_12 h_: 52 (22–102) MVC AUC_0–12 h_: 2,086 (1,477–4,372) h·ng/mL	SP	Single dose: MVC C12h: 22.3 MVC AUC_0–12 h_: 700 h·ng/mL Multiple dose MVC C_12 h_: 38 (18–74) AUC_0–12 h_: 1,123 (633–2,087) h·ng/mL	Single dose MVC C_12 h_: 0.98 AUC_0–12 h_: 0.45 Multiple dose MVC C_12 h_: 0.68 (0.48–0.95) AUC_0–12 h_: 0.56 (0.44–0.70)
Calcagno et al. (129)*	10	PWH	MVC + LPV/r	150 mg QD	MVC C_12 h_: 223 (103–312)	SP	MVC C_12 h_: 527 (234–852)	MVC C_12 h_: 2.9 (1–4)

^*a*^ Summary of studies that quantified male genital tract concentrations of contemporary PIs. Values are standardized to median (range) [†] or median (IQR)^ [*]^unless stated otherwise. Units are standardized to ng/mL unless stated otherwise.Pilcrow (¶) indicates values that were calculated from raw data and are standardized to median (IQR).

^*b*^ ARV, antiretroviral; AUC, area under the curve; BID, twice daily; BP, blood plasma; C_12 h_, concentration 12 h post-dose; LPV, lopinavir; MGT, male genital tract; MVC, maraviroc; PWH, people with HIV; PWOH, people without HIV; QD, once a day; /r, ritonavir as pharmacoenhancer; RAL, raltegravir; RPV, rilpivirine; SP, seminal plasma.

^*c*^ N/A, not reported in associated reference.

#### Maraviroc

In participants administered MVC—in combination with PIs, INSTIs, and PK boosters—the median observed SP:BP was 9.7 (113). Conversely, other studies including participants on MVC alone and in combination with PK-boosted PIs reported SP:BPs ranging from 0.35 to 2.9 at the end of the 12-h dosing interval (85, 111, 116). Two separate studies reported MVC SP protein-binding potential ranging from 3.6% to 24.8% versus 61% to 70% in BP, resulting in higher protein-unbound SP concentrations compared to BP (101, 128). HIV RNA was undetectable in one study (129). In contrast, other studies have found intermittent viral shedding despite effective MVC concentrations in the BP (101). Osborne et al. noted isolated viral shedding following the addition of MVC and RAL to a background regimen (70). Lastly, following a single dose of MVC in people without HIV, MVC concentrations were 157-fold higher in urethral discharge than in BP (130).

### Concluding thoughts about ARV exposure in the MGT

Across the class, NRTIs preferentially distribute into the SP. By contrast, median NRTI intracellular metabolite SMC:PBMCs were ≤1. Taken together, higher NRTI SP concentrations do not necessarily predict greater intracellular phosphorylation within SMCs. NNRTIs universally demonstrate non-preferential distribution into the MGT. The discussed PIs demonstrated poor penetration into the MGT, and similar trends were observed with INSTIs. The key exception was RAL.

**Fig 3:**
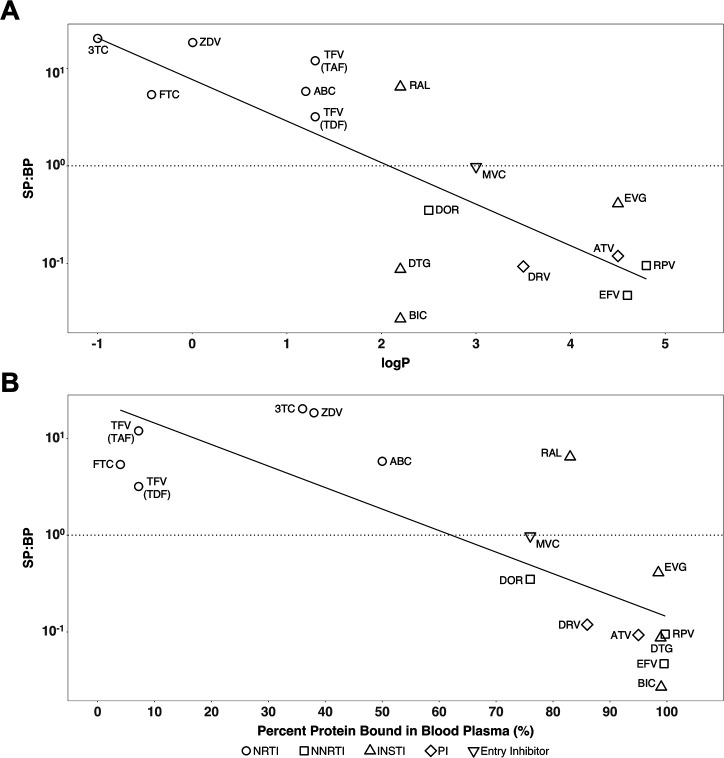
Seminal plasma-to-blood plasma ratio (SP:BP) versus partition coefficient (logP) (**A**) and SP:BP versus percent protein bound in blood plasma (**B**). Each data point in these plots represents the median SP:BP ratio for each antiretroviral from the studies described in the accompanying Tables 1 to 6. The diagonal line represents the trendline, indicating the inverse relationship between SP:BP and logP, and SP:BP and percent protein bound in blood plasma. Abbreviations: 3TC, lamivudine; ABC, abacavir; ATV, atazanavir; BIC, bictegravir; DOR, doravirine; DRV, darunavir; DTG, dolutegravir; EFV, efavirenz; EVG, elvitegravir; FTC, emtricitabine; INSTI, integrase strand transfer inhibitor; MVC, maraviroc; NNRTI, non-nucleoside reverse transcriptase inhibitor; NRTI, nucleoside reverse transcriptase inhibitor; PI, protease inhibitor; RAL, raltegravir; RPV, rilpivirine; TAF, tenofovir alafenamide; TDF, tenofovir disoproxil fumarate; TFV, tenofovir; ZDV, zidovudine.

The ARVs discussed herein demonstrated an inverse relationship between the degree of lipophilicity (logP) and the SP:BP, which is shown in Fig. 3A. This inverse relationship may be driven by the tendency for lipophilic drugs to demonstrate high BP protein binding relative to hydrophilic drugs (131). Additionally, SP is aqueous, which may generally limit the solubility and accumulation of lipophilic ARVs in this matrix (132). Unsurprisingly, a higher degree of protein binding in BP is associated with lower SP:BPs, a relationship shown in Fig. 3B. As described above, lower SP:BPs were observed for NNRTIs, PIs, BIC, and DTG, all of which have moderate-to-high (e.g. >85%) protein binding in BP.

## PHARMACOLOGICAL GAPS AND FUTURE RESEARCH OPPORTUNITIES

### Long-acting injectables

Long-acting injectables (LAIs) are novel formulations of ARVs that provide unique opportunities for understanding MGT pharmacology throughout the dosing interval. LAIs are dosed on the order of months, allowing for multiple semen samples to be collected during a single dosing interval from the same participant. This will allow researchers to obtain more robust SP concentration-time curves for subsequent PK analyses.

#### Cabotegravir/Rilpivirine

Cabotegravir/RPV (CAB/RPV) is approved as a monthly or bimonthly intramuscular injection with an optional oral lead-in period for treatment in PWH who have achieved viral suppression with a stable oral ART regimen (133). CAB alone is approved for pre-exposure prophylaxis (PrEP) (134).

In a study of 16 men initiating bimonthly CAB/RPV, Fernández et al. quantified CAB and RPV SP concentrations at the end of the dosing interval four months after initiation (135). Total CAB SP:BP was 0.02 with a median SP concentration of 23.3 ng/mL (135). However, CAB protein-binding potential in the SP (56.6%) was lower than the BP (>99.9%), resulting in a protein-unbound SP:BP of 13.0 (135). Despite non-preferential penetration, protein-unbound CAB concentrations in SP remained 90-fold higher than the *in vitro* unbound EC_50_ (0.23 ng/mL) (135, 136). Further studies are warranted to understand the magnitude of CAB penetration into the MGT throughout the extended dosing interval.

Similar to oral RPV administration, median RPV SP:BP following IM administration was low (0.08) at the end of the dosing interval (135). RPV SP protein-binding potential was 97.0% (135), lower than 99.5% following oral RPV administration reported in a previous study (101). In contrast to CAB, RPV demonstrated a protein-unbound SP:BP of 1.8, and protein-unbound SP concentrations were 4.5-fold lower than the *in vitro* EC_50_ (0.27 ng/mL) (105, 135). This suggests RPV may not maintain effective concentrations in SP following intramuscular injection, although additional studies are necessary to evaluate RPV penetration throughout the dosing interval to confirm these findings.

#### Lenacapavir

Lenacapavir (LEN) is administered as a subcutaneous injection every 6 months following one of two oral initiation regimens (137). LEN is a first-in-class capsid inhibitor initially approved as treatment for patients with multidrug-resistant HIV (137). In 2025, the United States Food & Drug Administration approved LEN for PrEP following data from Phase 3 studies that demonstrated superiority over TDF/FTC (138, 139).

LEN has a high logP (6.4) and is highly protein-bound in BP (99.8%) (45). These properties, along with the trends seen in Fig. 3A and B, suggest that LEN will minimally penetrate the MGT. Additionally, LEN is a substrate of P-gp and CYP3A, both of which would prevent accumulation in the MGT (45). Further studies are needed to characterize the MGT distribution of LEN throughout the dosing interval to confirm these hypotheses.

### Investigational drugs

#### Islatravir

Islatravir (ISL)—also referred to as (4′-ethynyl-2-fluoro-2′-deoxyadenosine; EFdA)—is a first-in-class nucleoside reverse transcriptase translocation inhibitor currently undergoing Phase 3 clinical trials (140). Similar to NRTIs, ISL must be phosphorylated intracellularly by deoxycytidine kinase to the active metabolite ISL triphosphate (ISL-TP; EFdAtp) (140). Kovarova et al. demonstrated in HIV-infected bone marrow/liver/thymus humanized mice that ISL effectively reduces viral load in the testes, epididymis, seminal vesicles, prostate, and penis (141). Additionally, ISL effectively prevented HIV acquisition following penile challenges on days 1, 4, and 8 after treatment initiation (141). Together, these indicate that ISL and ISL-TP concentrations may be therapeutic in the MGT, but additional clinical studies are warranted.

#### Broadly neutralizing antibodies

Broadly neutralizing antibodies (bNAbs) represent a new approach toward HIV treatment and prevention with unique pharmacological principles (142). bNAbs neutralize HIV-infected cells and recruit immune cells for effective clearance (143). Half-lives of bNAbs span from weeks to months, making them ideal candidates to be combined with other LAIs for treatment (143). Due to their large molecular weight (150 kilodaltons), bNAbs struggle to cross tight anatomical barriers (144). The penetration of bNAbs across the BTB is unexplored, but penetration across a similarly tight barrier—blood-brain barrier—is poor (145). Given this poor penetration, bNAbs may not cross the similarly tight anatomical barriers of the MGT, limiting their accumulation in the testes and prostate.

### Computational approaches to study site of action-specific pharmacology

#### PK/pharmacodynamic modeling

PK/pharmacodynamic (PK/PD) modeling is a powerful tool for understanding drug distribution and efficacy in the MGT. Population PK models have been developed to understand differences in NRTI concentrations between BP and SP (92, 146) and the association between NRTI and phosphorylated metabolites in SP and SMCs, respectively (76, 147).

Valade et al. built the first population PK model to simultaneously describe FTC exposure in BP and SP in men with HIV, to identify an association between FTC SP exposure and SP viral load (76). Authors estimated the FTC BP-to-SP transfer rate constant, defined as the rate at which FTC distributes into SP from the central compartment (76). While covariates were examined, none statistically explained the high interindividual variability for this parameter (76).

Valade et al. built a similar model for TFV exposure in BP and SP following TDF administration, but additionally included genetic polymorphisms as a covariate to explain TFV transfer rate into SP (146). While genetic polymorphisms were not significantly associated with the TFV transfer rate, the polymorphism for MRP4—of which TFV is a substrate—had the highest significance (31, 146). In both of these studies, SP HIV viral loads were detectable (>100 copies/mL) for ~10% of participants, but these were not statistically significantly associated with SP AUC_0-24h_ or SP:BP AUC_0-24h_ for either ARV (76, 146). Notably, these models had not examined intracellular FTCtp and TFVdp exposure, which may be more associated with detectable SP HIV viral load.

Toward these efforts, Greene et al. developed population PK models to inform our mechanistic understanding of TFV PK in SP and BP, as well as TFVdp PK in PBMCs and SMCs following TAF and TDF administration (147). These models were created after noncompartmental PK analyses revealed similar TFV SP and TFVdp SMC exposure regardless of formulation, despite significantly lower TFV BP and significantly higher TFVdp PBMC exposure following TAF compared to TDF (147). In the models, authors included specific PK compartments representing TFVdp in PBMCs and SMCs following the administration of both TFV prodrug formulations (147). By simultaneously modeling TFV and TFVdp PK, the authors were able to estimate descriptive parameters (e.g., TFV BP-to-SP transfer and TFVdp elimination rate), revealing that TFV SP and TFVdp SMC disposition differ based on formulation and are not driven by TFV BP exposure (147). This study demonstrated how population PK modeling can help to overcome limitations of noncompartmental PK analyses and increase our mechanistic understanding of ARV disposition in the MGT.

Finally, population PK models were developed to describe the PK of 3TC and ZDV in SP and BP and 3TCtp and ZDVtp in PBMCs and SMCs (92). These models sought to understand why 3TCtp and ZDVtp exhibit SMC:PBMCs ≤1 despite 3TC and ZDV preferentially distributing into SP (92). The models showed that 3TCtp demonstrated different intracellular formation rates and that both 3TCtp and ZDVtp had different intracellular elimination rates in PBMCs compared to SMCs, explaining the discrepancy between SP:BP and SMC:PBMC for these NRTIs (92).

In addition to population PK modeling, physiologically based PK (PBPK) modeling can provide organ-specific predictions of ARV distribution by integrating physicochemical properties of ARVs alongside anatomy and physiology (148). This approach predicts drug-drug interactions and simulates tissue exposure in difficult-to-sample compartments (148). For instance, PBPK models have provided insight into the PK of flutamide—a drug used for the treatment of metastatic prostate cancer—in the prostate and testes (149, 150). PK/PD approaches can be similarly employed with newer ARVs and LAIs to help answer clinically relevant research questions, such as drug-drug interactions and missed or delayed injections.

#### Multiomics techniques

Multiomics is a hypothesis-generating discipline that enables the simultaneous analysis of thousands of molecules to gain an understanding of biological mechanisms at a systems level (151). These include lipids (termed lipidomics), proteins (proteomics), and microbes (microbiomics) (152). Multiomics analyses can be applied to the MGT to better understand the biological factors influencing HIV persistence. These approaches can collectively clarify the interplay between HIV viral dynamics, local microbiome, lipid composition, inflammation, and protein content in the MGT (152). Studies in SP may provide insight into the biological underpinnings of HIV and ART affecting inflammatory signaling within the MGT.

Proteomics can identify site-specific biomarkers of inflammation (e.g., prostate-specific antigen) within the SP, providing detailed quantitative data regarding the unique immunological environments of SP compared to BP (153). Additionally, as had been performed previously, targeted proteomics can quantify DMEs and transporters at specific sites of action to understand modulators of ARV distribution throughout the MGT (30, 154).

The penile microbiome of uncircumcised men includes diverse anaerobic bacteria that are associated with local inflammation and increased risk of HIV acquisition (155). Additionally, increased bacterial diversity in the semen of men with HIV is directly associated with increased inflammatory cytokine concentrations and increased viral shedding in the semen (156). These findings highlight the potential utility of microbiomics as a discipline for understanding local inflammation in the MGT.

Understanding the site-specific inflammatory milieu in PWH may elucidate how inflammation shapes ARV distribution and viral persistence in the MGT (59). Furthermore, integrating these robust systems-level data with PK/PD modeling will allow researchers to answer questions towards precision medicine initiatives (157). These questions include the following: (i) How do patient-specific inflammatory and/or microbiome profiles influence penetration of ARVs into the MGT? and (ii) How do MGT microbiomes and inflammation impact ARV efficacy at this site of action?

## CONCLUDING THOUGHTS

Our review highlights the interclass and intraclass pharmacological profiles of ARVs in the MGT. These profiles are influenced by numerous factors, including BP protein binding, physicochemical properties, active and passive transport mechanisms, and metabolism within MGT organs. While low MGT drug concentrations could theoretically permit local replication or intermittent shedding despite undetectable plasma HIV RNA, the clinical significance remains uncertain. The preponderance of epidemiological and clinical evidence indicates that sustained plasma viral suppression correlates strongly with the absence of sexual transmission.

The literature reviewed herein includes observational and small PK studies that examine SP drug concentrations and occasional reports of seminal HIV RNA detection despite undetectable plasma RNA, but these findings have not consistently translated into demonstrable transmission events in the context of maintained systemic viral suppression. Consequently, while genital tract pharmacology is scientifically relevant and could inform future drug optimization (e.g., ensuring adequate drug exposure in key reservoirs), current public health recommendations that base transmission risk primarily on durable plasma viral suppression remain appropriate.

Nevertheless, the MGT is a compartment highly relevant to viral transmission, highlighting the importance of understanding MGT pharmacology during clinical situations that lead to variable systemic drug exposure, including treatment initiation, changes in treatment regimens, and imperfect adherence. We therefore suggest acknowledging the potential mechanistic role of genital tract pharmacology and recommending further prospective studies correlating MGT drug concentrations, local viral replication/shedding, and transmission outcomes to further inform guidelines.

LAIs have ushered in a new era of HIV treatment and prevention, introducing promising alternatives alongside unique pharmacological challenges. Future research efforts should aim to describe their pharmacology within the MGT, especially as additional ARVs are investigated. Understanding ARV pharmacology in the MGT and other tissues will be essential for developing curative strategies, as adequate tissue penetration is necessary to facilitate depletion of the latent tissue reservoir. Through integration of established and emerging computational approaches, the field can answer clinically relevant questions related to the complex interplay between the factors affecting ARV penetration and efficacy in the MGT and advance precision medicine initiatives.
